# Efficacy and tolerability of short-term duloxetine treatment in adults with generalized anxiety disorder: A meta-analysis

**DOI:** 10.1371/journal.pone.0194501

**Published:** 2018-03-20

**Authors:** Xinyuan Li, Lijun Zhu, Chunkui Zhou, Jing Liu, Heqian Du, Chenglin Wang, Shaokuan Fang

**Affiliations:** 1 Department of Neurology, Neuroscience Centre, the First Teaching Hospital of Jilin University, Changchun, China; 2 China-Japan Union Hospital of Jilin University, Changchun, China; University of Catanzaro, ITALY

## Abstract

**Objective:**

To investigate the efficacy and tolerability of duloxetine during short-term treatment in adults with generalized anxiety disorder (GAD).

**Methods:**

We conducted a comprehensive literature review of the PubMed, Embase, Cochrane Central Register of Controlled Trials, Web of Science, and ClinicalTrials databases for randomized controlled trials(RCTs) comparing duloxetine or duloxetine plus other antipsychotics with placebo for the treatment of GAD in adults. Outcome measures were (1) efficacy, assessed by the Hospital Anxiety and Depression Scale(HADS) anxiety subscale score, the Hamilton Rating Scale for Anxiety(HAM-A) psychic and somatic anxiety factor scores, and response and remission rates based on total scores of HAM-A; (2) tolerability, assessed by discontinuation rate due to adverse events, the incidence of treatment emergent adverse events(TEAEs) and serious adverse events(SAEs). Review Manager 5.3 and Stata Version 12.0 software were used for all statistical analyses.

**Results:**

The meta-analysis included 8 RCTs. Mean changes in the HADS anxiety subscale score [mean difference(MD) = 2.32, 95% confidence interval(CI) 1.77–2.88, P<0.00001] and HAM-A psychic anxiety factor score were significantly greater in patients with GAD that received duloxetine compared to those that received placebo (MD = 2.15, 95%CI 1.61–2.68, P<0.00001). However, there was no difference in mean change in the HAM-A somatic anxiety factor score (MD = 1.13, 95%CI 0.67–1.58, P<0.00001). Discontinuation rate due to AEs in the duloxetine group was significantly higher than the placebo group [odds ratio(OR) = 2.62, 95%CI 1.35–5.06, P = 0.004]. The incidence of any TEAE was significantly increased in patients that received duloxetine (OR = 1.76, 95%CI 1.36–2.28, P<0.0001), but there was no significant difference in the incidence of SAEs (OR = 1.13, 95%CI 0.52–2.47, P = 0.75).

**Conclusion:**

Duloxetine resulted in a greater improvement in symptoms of psychic anxiety and similar changes in symptoms of somatic anxiety compared to placebo during short-term treatment in adults with GAD and its tolerability was acceptable.

## Introduction

Generalized anxiety disorder (GAD) is one of the most common anxiety disorders in adults. Epidemiological surveys estimate the lifetime prevalence of GAD at 2.8–6.2% and the 12 month prevalence at 0.2–4.3% [1]. In the National Comorbidity Replication Survey, the 12-month prevalence of GAD was approximately 12% in adults over the age of 55 years[2].

GAD is characterized by pervasive, excessive, and difficult-to-control worry [3], and the presence of psychic and somatic symptoms [4]. Psychic symptoms include restlessness, difficulty concentrating, irritability, and feeling keyed up [5]. Somatic symptoms include muscle tension, sleep disturbance, gastrointestinal dysfunction, cardiovascular disease, and impairment in other organ systems [6]. The presence and severity of psychic and somatic anxiety symptoms are commonly assessed using the Hamilton Rating Scale for Anxiety (HAM-A) psychic and somatic anxiety factor scores [7–8].

First-line pharmacotherapy for GAD involves selective serotonin reuptake inhibitors (SSRIs), serotonin norepinephrine reuptake inhibitors (SNRIs), and pregabalin [9]. Efficacy and tolerability of these pharmacological agents are measured by improvement in psychic and somatic anxiety symptoms [10] and the occurrence of treatment-emergent adverse events (TEAEs) (nausea, constipation, dry mouth, dizziness, somnolence), respectively.

Duloxetine is an SNRI that was approved by the Food Administration (FDA) in 2007 as an efficacious and well-tolerated first-line treatment option for GAD [5,11]. Several randomized controlled trials (RCTs) have also been conducted to examine its efficacy for improving symptoms of psychic and somatic anxiety and tolerability in patients with GAD [5,12–16]; however, to the author’s knowledge, there are no comprehensive meta-analyses investigating these. In addition, findings from meta-analyses in therapeutic areas such as GAD where there are multiple first-line treatment options can facilitate clinical decision-making for physicians selecting medications. Therefore, we conducted a meta-analysis to investigate the efficacy and tolerability of duloxetine during short-term treatment in adults with GAD.

## Methods

This meta-analysis was performed according to the protocol provided as supporting material(S1 File), and was reported as recommended by the Preferred Reporting Items for Systematic Reviews and Meta-Analyses (PRISMA) guidelines [17](S2 File).

### Search strategy

Two review authors (Xinyuan Li, Lijun Zhu) independently searched the PubMed, Embase, Web of Science, Cochrane Center Register of Controlled Trials (CENTRAL), and ClinicalTrials databases from inception to October 4, 2017 using the search terms (duloxetine OR LY248686 OR cymbalta) AND (generalized anxiety disorder OR GAD).The detailed search strategy was available (S3 File). Searches were limited to RCTs and publications in the English language. Manual searches of the reference lists for all relevant articles were conducted, and corresponding authors of some trials were contacted for missing information. The search was updated on November 10, 2017 using the same strategy.

### Inclusion and exclusion criteria

Inclusion criteria were: (1) population: ≥18 years of age with a diagnosis of GAD according to the Diagnosis and Statistical Manual of Mental Disorders, Fourth Edition (DSM-IV) [18]; (2) study design: placebo-controlled RCTs; (3) intervention: duloxetine or duloxetine plus other antipsychotics for<6 months; (4) outcomes: efficacy and tolerability outcomes.

Trials were excluded if they included patients with: (1) DSM-IV diagnosis of major depressive disorder, bipolar disorder, or other psychotic disorders within the past 6 months; (2) use of any neuroleptic, antidepressant, or anxiolytic agent in the two weeks before data collection at baseline; (3) history of alcohol or any psychoactive substance abuse or dependence (as defined by DSM-IV) within the past 6 months; (4) risk of suicide; (5) previous treatment with duloxetine before randomization. Trials were also excluded if they did not report HAM-A psychic and somatic anxiety factor scores.

### Data extraction

Two review authors (Xinyuan Li, Lijun Zhu) independently assessed eligible trials. The full text of potentially relevant trials was examined and the following data were extracted: first author’s name, year of publication, study design, patient population, sample, age, sex distribution, intervention, treatment duration, and efficacy and tolerability outcomes using the last-observation-carried-forward (LOCF) approach.

Disagreements were resolved by discussion with a third review author until consensus was reached.

### Outcomes and definitions

The primary efficacy outcome was mean change in the Hospital Anxiety and Depression Scale(HADS) anxiety subscale score from baseline to endpoint. The key secondary efficacy outcomes were mean changes in HAM-A psychic and somatic anxiety factor scores. Other secondary efficacy outcomes were response and remission rates. The response was defined as ≥50% reduction from baseline in the HAM-A total score and remission was defined as HAM-A total score ≤7 at endpoint. Tolerability outcomes were discontinuation rate due to AEs and commonly reported TEAEs including nausea, constipation, dry mouth, dizziness, and somnolence.

### Quality assessment

Two review authors (Xinyuan Li, Lijun Zhu) independently assessed the risk of bias in included trials using the Cochrane Collaboration’s Risk of Bias Tool [19]. Reviewers examined seven domains including: random sequence generation, allocation concealment, blinding of outcome assessment, blinding of participants and personnel, incomplete outcome data, selective reporting, and other bias. Risk of bias was categorized as low, high, or unclear.

Disagreements about quality assessment were resolved by discussion with a third review author until consensus was reached.

### Statistical analysis

Statistical analysis was performed using Review Manager 5.3 (Cochrane Collaboration, London, UK). All analyses were conducted on the intent-to-treat (ITT) populations. Mean differences (MDs) with 95% confidence intervals (CIs) were calculated for continuous variables, and odds ratios (ORs) with 95% CIs were calculated for dichotomous variables. A random-effects model was used to pool studies with substantial heterogeneity, as determined by the chi-squared test (P<0.05) and the inconsistency index (I^2^ ≥ 50%) [20–21]. Publication bias was assessed with funnel plots and the Begg’s/Egger’s test[22–23] using Stata 12.0 software. We also conducted sensitivity analyses to evaluate the stability of the outcomes. The significance of the pooled estimates was determined by the Z statistic; statistical significance was set at P<0.05[24].

## Results

### Characteristics of included studies

**Fig 1:**
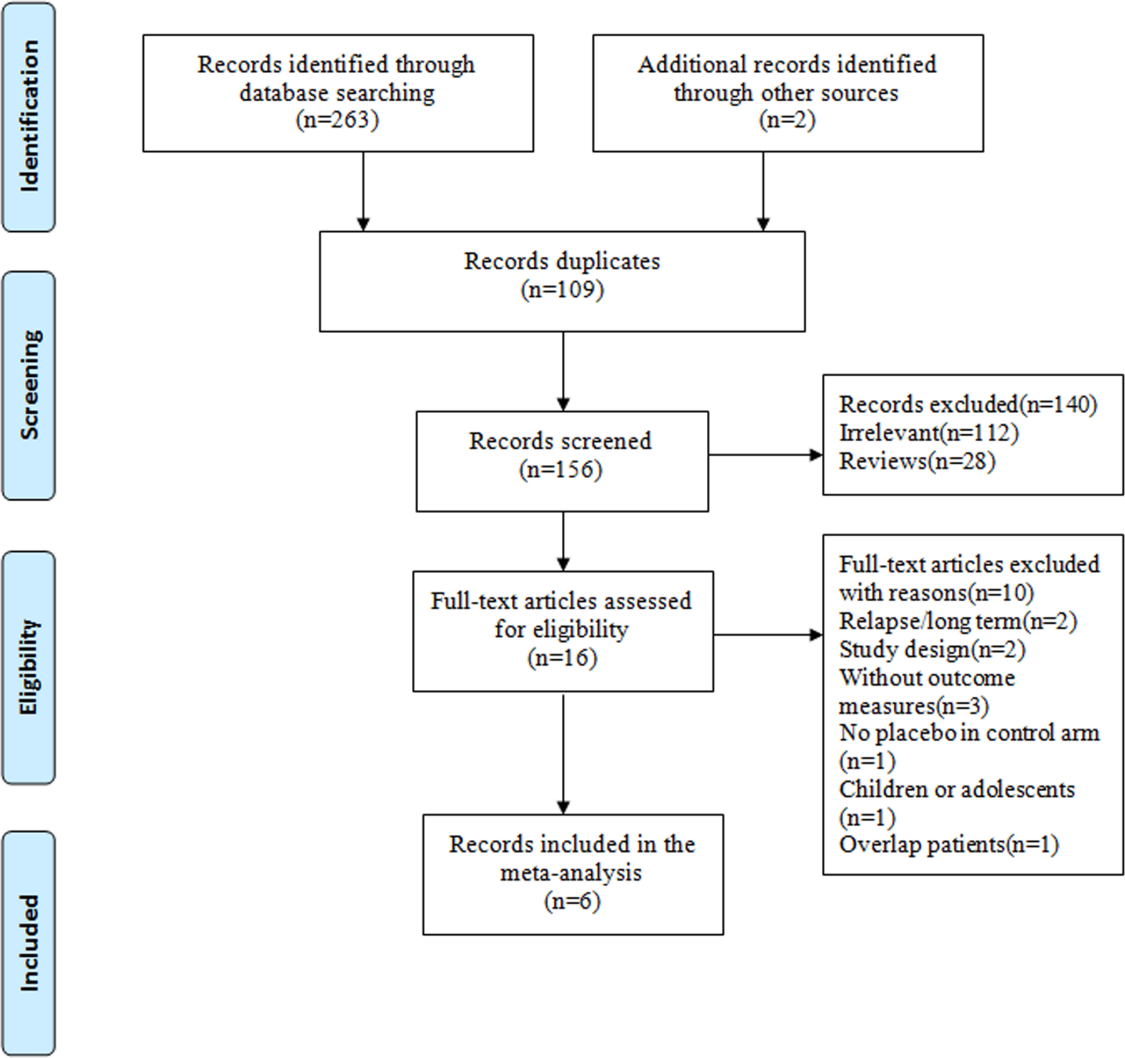
Flow chart of the literature search.

The searches identified a total of 265 articles. Titles and abstracts were screened, and 249 articles were excluded. Among these, 112 were irrelevant, 109 were duplicates, and 28 were reviews. The full text of 16 articles was examined, and 10 articles were excluded due to inappropriate study design, study population, or outcome measures. Finally, 6 articles that described 8 RCTs were considered eligible for inclusion in our meta-analysis (Fig 1).

The characteristics of the included trials were shown in Table 1. The 8 RCTs were conducted between 2007 and 2014. The trials included 2,399 adult patients with moderate intensity GAD, defined as a HADS anxiety subscale score≥10 (higher score represents great disease severity; max score = 21). Among the included patients, 1,161 were treated with duloxetine, and 1,238 were treated with placebo. Five trials lasted 10 weeks, two trials lasted 9 weeks, and one trial lasted 15 weeks. Three trials administered fixed doses of duloxetine at 20mg/day, 60mg/day, and 120mg/day; four trials administered flexible doses of 60-120mg/day, and one trial administered a flexible dose of 30-120mg/day.

**Table 1:** Characteristics of included RCTs.

Study	Design	Sample size(N)	Male/ female (N)	Diagnosis criteria	Treatment/ control	Age years (mean±SD)	DLX Dose(mg/d)	Duration (weeks)	Baseline HADS anxiety subscale (mean±SD)	Entry score	Mean change of psychic factor score^a^ (mean±SD)	Mean change of somatic factor score^a^ (mean±SD)	Location
**Hartford 2007[12]**	Double- blind	323	120/203	GAD, DSM-IV	DLX/PBO	40.4±13.6/ 41.9±14.2	60–120	10	14.3±2.8/ 13.9±3.2	≥10	7.01±5.35/ 5.13±5.08	4.74±4.33/ 4.08±4.19	the US
**Koponen 2007a[5]**	Double- blind	343	118/225	GAD, DSM-IV	DLX/PBO	43.1±12.9/ 44.1±13.4	60	9	13.1±3.7/ 13.3±3.9	≥10	7.57/4.53	5.19/3.82	7 countries
**Koponen 2007b[5]**	Double- blind	345	105/240	GAD, DSM-IV	DLX/PBO	44.1±12.6/ 44.1±13.4	120	9	12.9±3.9/ 13.3±3.9	≥10	7.15/4.53	5.33/3.82	7 countries
**Rynn 2008[13]**	Double- blind	327	125/202	GAD, DSM-IV	Dlx/PBO	42.2±13.9/ 41.0±14.2	60–120	10	12.5±3.7/ 12.5±3.5	≥10	5.33/3.33	2.81/2.54	the US
**Nicolini 2009a[14]**	Double- blind	246	-/-	GAD, DSM-IV	DLX/PBO	42.8	20	10	-/-	≥10	8.1±5.5/ 6.0±5.1	6.6±4.6/ 5.5±3.8	8 countries
**Nicolini 2009b[14]**	Double- blind	314	-/-	GAD, DSM-IV	DLX/PBO	42.8	60–120	10	-/-	≥10	8.7±4.9/ 6.0±5.1	6.6±4.9/ 5.5±3.8	8 countries
**Wu 2011[15]**	Double- blind	210	104/106	GAD, DSM-IV	DLX/PBO	37.3±11.9/ 38.0±12.0	60–120	15	12.8±2.9/ 13.5±3.0	≥10	8.05±4.68/ 6.61±4.75	6.26±4.05/ 5.14±4.04	China
**Alaka 2014[16]**	Double- blind	291	65/226	GAD, DSM-IV–TR	DLX/PBO	71.4±5.4/ 71.7±5.0	30–120	10	13.9±3.1/ 13.6±3.3	≥10	8.6±4.9/ 6.2±4.7	7.3±3.7/ 5.6±4.7	9 countries

DLX, duloxetine; PBO, placebo; HADS, Hospital Anxiety Depression Scale; SD, standard deviation; LOCF, last observation carried forward; -, not applicable.

^**a**^ endpoint measure(LOCF).

### Quality assessment

**Fig 2:**
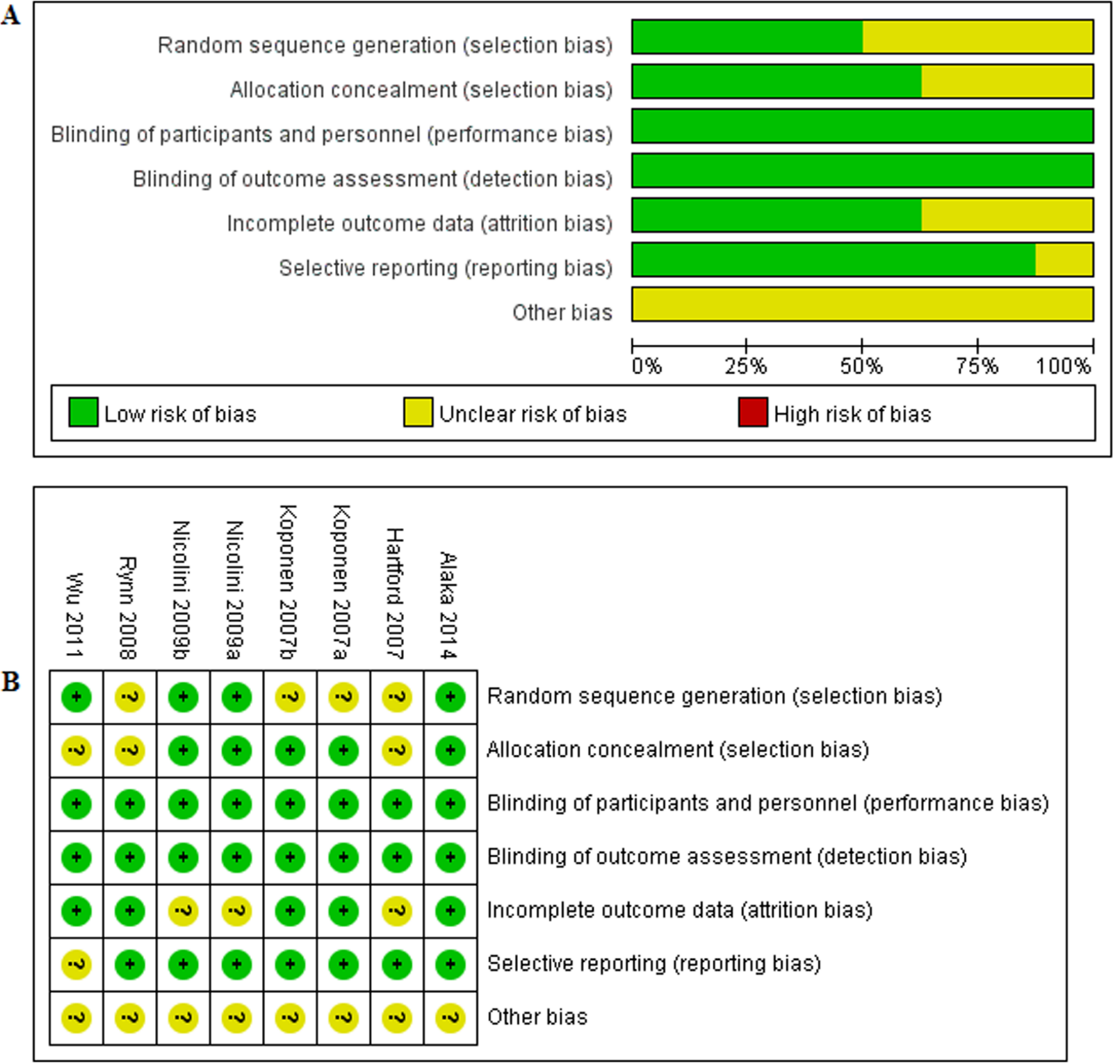
Assessment of the quality of included studies. (A) risk of bias graph. (B) risk of bias summary.

Overall, risk of bias in the included RCTs was low or unclear (Fig 2). Risk of bias across studies was shown in Fig 2A and risk of bias in individual studies was shown in Fig 2B.

### Publication bias

**Fig 3:**
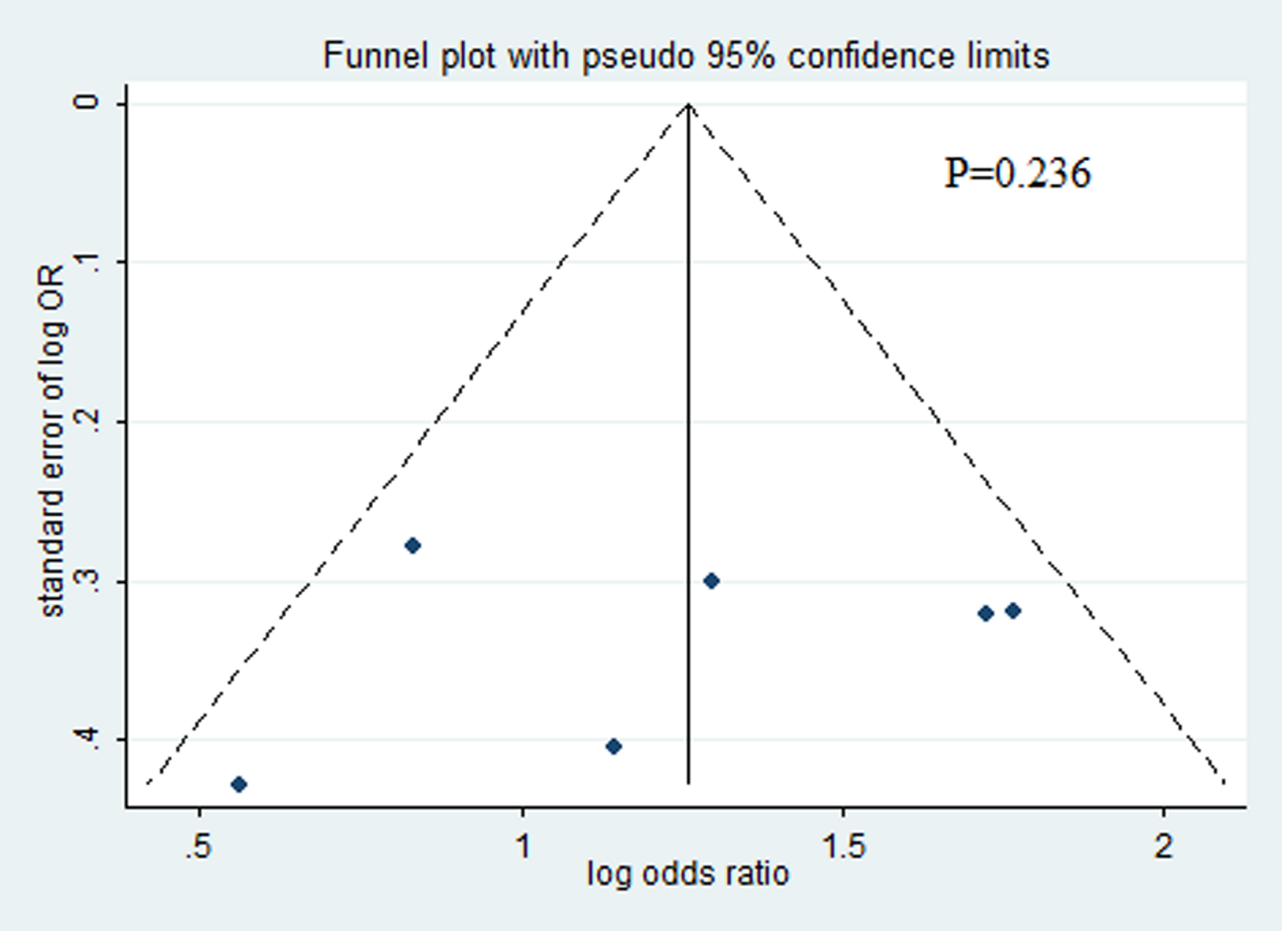
Funnel plot of publication bias.

Visual inspection of the funnel plot, and the Begg’s/Egger’s test revealed no significant publication bias(P = 0.236)(Fig 3).

### Outcomes

#### Primary efficacy outcome

**Fig 4:**
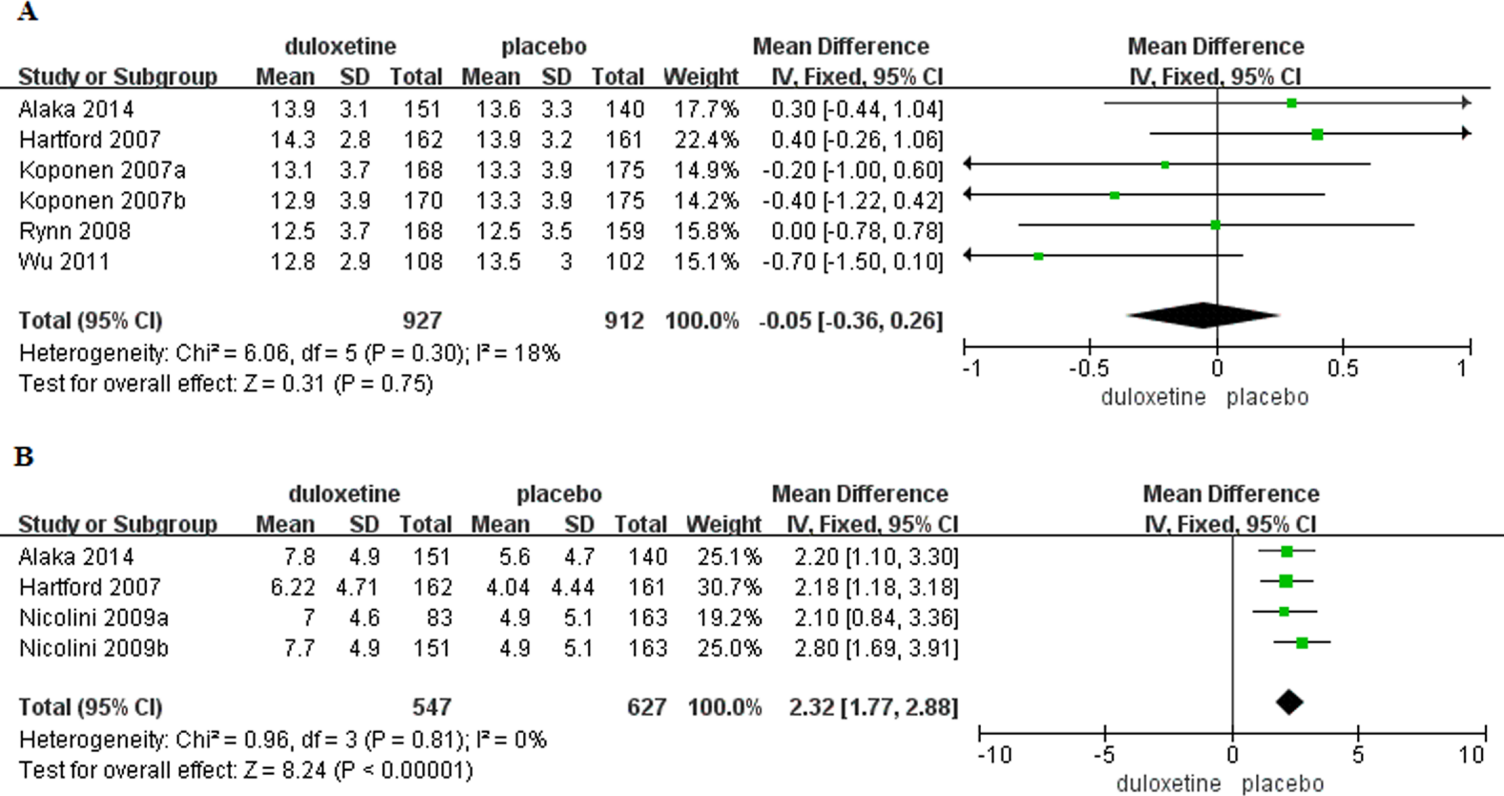
Forest plots of HADS anxiety subscale score. (A) Baseline. (B) Mean change with treatment.

Baseline HADS anxiety subscale score was reported in six trials [5,12–13,15–16](duloxetine, n = 927; placebo, n = 912). There was no significant difference in baseline HADS anxiety subscale score in patients with GAD that received duloxetine compared to those that received placebo (MD = -0.05, 95%CI -0.36–0.26, P = 0.75)(Fig 4A). Change in HADS anxiety subscale score from baseline to the end of the study was reported in four trials[12,14,16] (duloxetine, n = 547; placebo, n = 627). Mean change in the HADS anxiety subscale score was significantly greater in patients with GAD that received duloxetine compared to those that received placebo (MD = 2.32, 95%CI 1.77–2.88, P<0.00001)(Fig 4B). There was no evidence of significant heterogeneity (P = 0.81, I^2^ = 0%).

#### Secondary efficacy outcomes

**Fig 5:**
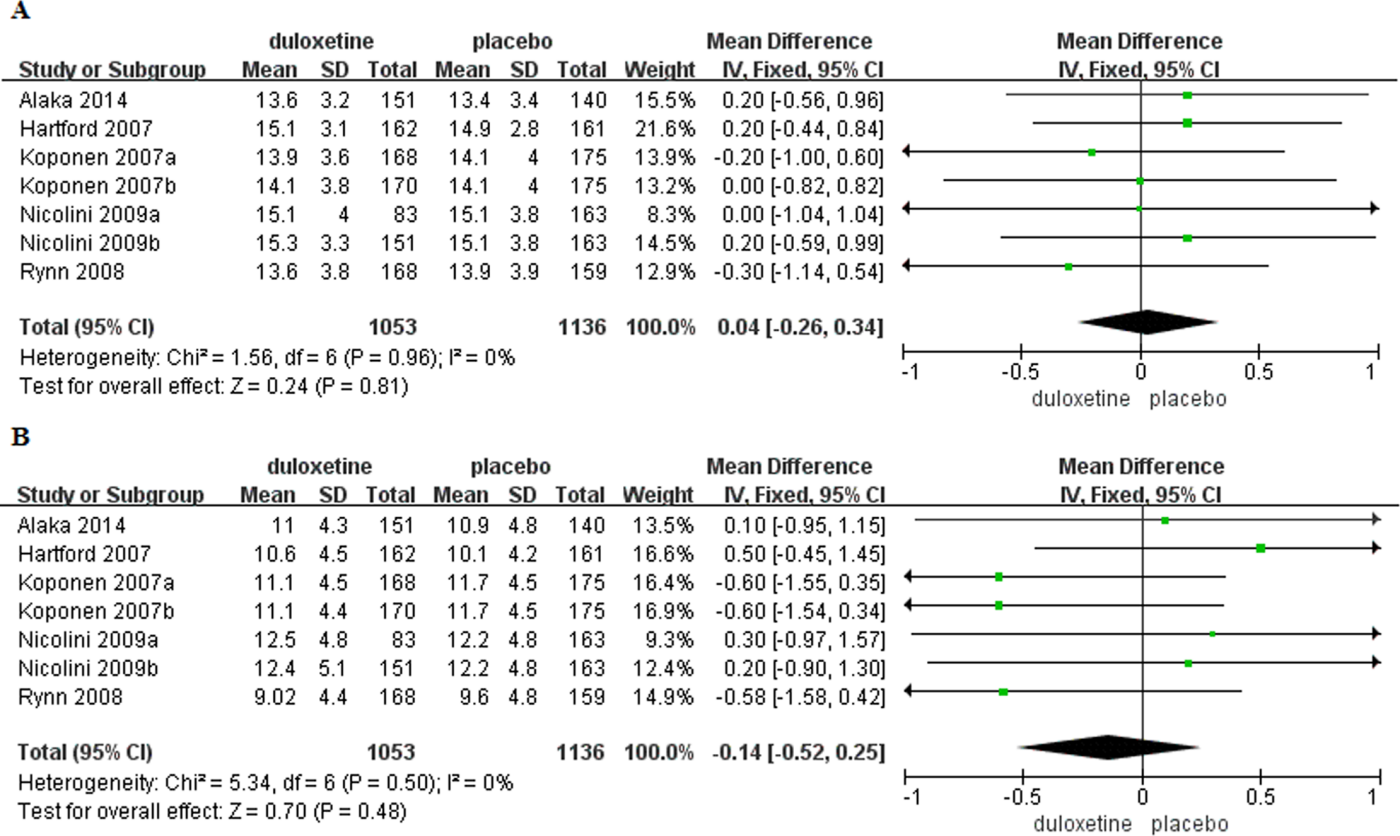
Forest plots of HAM-A psychic and somatic anxiety factor scores. (A) Baseline psychic anxiety factor score. (B) Baseline somatic anxiety factor score.

**Fig 6:**
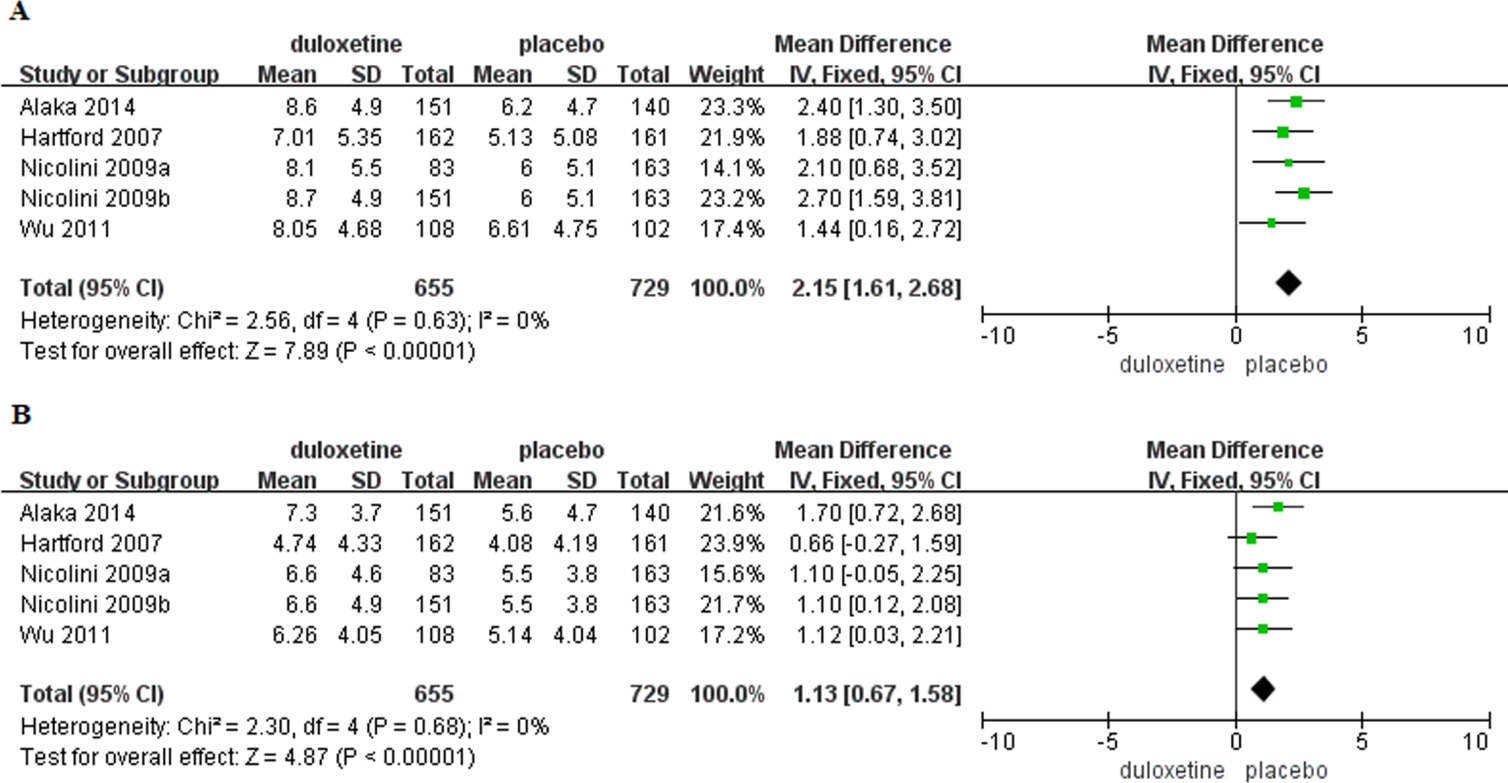
Mean changes in HAM-A psychic and somatic anxiety factor scores. (A) Mean change in psychic anxiety factor score with treatment. (B) Mean change in somatic anxiety factor score with treatment.

Baseline HAM-A psychic and somatic anxiety factor scores were reported in seven trials [5,12–13,14,16](duloxetine, n = 1,053; placebo, n = 1,136). There were no significant differences in baseline HAM-A psychic and somatic anxiety factor scores in patients with GAD that received duloxetine compared to those that received placebo (psychic anxiety: MD = 0.04, 95%CI -0.26–0.34, P = 0.81; somatic anxiety: MD = -0.14, 95%CI -0.52–0.25, P = 0.48)(Fig 5). Changes in HAM-A psychic and somatic factor scores from baseline to the end of the study were reported in five trials[12,14–16](duloxetine, n = 655; placebo, n = 729). Mean change in HAM-A psychic anxiety factor score was significantly greater in patients with GAD that received duloxetine compared to those that received placebo (MD = 2.15, 95%CI 1.61–2.68, P<0.00001), but there was no significant difference in mean change in somatic anxiety factor score (MD = 1.13, 95%CI 0.67–1.58, P<0.00001)(Fig 6). There was no evidence of significant heterogeneity (psychic anxiety: P = 0.63, I^2^ = 0%; somatic anxiety P = 0.68, I^2^ = 0%). Response and remission rates were reported in eight trials[5,12–16] (duloxetine, n = 1,160; placebo, n = 1,236) and were both significantly higher in the duloxetine group as compared with placebo(response: OR = 2.22, 95%CI 1.88–2.62, P<0.00001, I^2^ = 44%; remission: OR = 1.99, 95%CI 1.66–2.39, P<0.00001, I^2^ = 48%)(S1 Fig).

#### Tolerability

**Fig 7:**
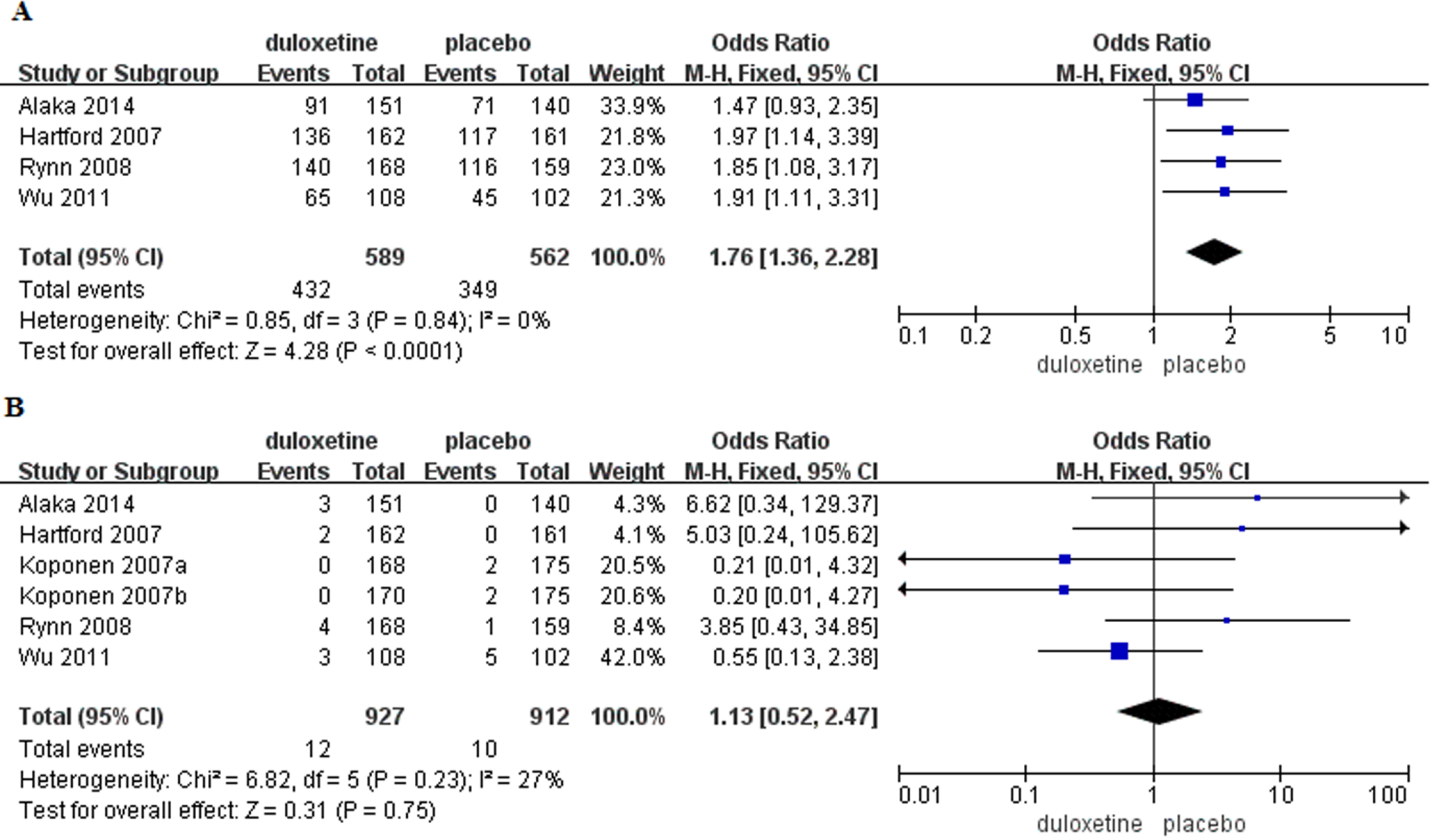
Forest plots of tolerability. (A) Incidence of any AE. (B) Incidence of SAEs.

Discontinuation due to AEs was reported in eight trials[5,12–16](duloxetine, n = 1,169; placebo, n = 1,252)(S2 Fig) and the pooled rate in the duloxetine group was significantly higher than the placebo group (OR = 2.62, 95%CI 1.35–5.06, P = 0.004). Heterogeneity was detected (I^2^ = 74%, P = 0.0003), thus, a random-effects model was used. Overall incidence of TEAEs (any AE) was reported in four trials[12–13,15–16] (duloxetine, n = 589; placebo, n = 562). The incidence of any AE was significantly increased in patients with GAD that received duloxetine compared to those that received placebo (OR = 1.76, 95%CI 1.36–2.28, P<0.0001)(Fig 7A) There was no evidence of significant heterogeneity (P = 0.84, I^2^ = 0%). There was no significant difference in the incidence of serious adverse events (SAEs) between the treatment groups (duloxetine, n = 927; placebo, n = 912; OR = 1.13, 95%CI 0.52–2.47, P = 0.75)(Fig 7B).

The most frequently reported TEAEs included nausea (mild, moderate, severe) dry mouth, dizziness, somnolence, decreased appetite, and decrease in libido[25]. The incidence of nausea, constipation, dry mouth, dizziness, and somnolence was significantly increased in patients with GAD that received duloxetine compared to those that received placebo (Table 2). For decrease in libido, one study reported no significant difference between treatment groups (OR = 2.19, 95%CI 0.66–7.27), while another reported that sexual dysfunction was significantly higher in patients with GAD that received duloxetine (OR = 11.66, 95%CI 1.49–91.37).

**Table 2:** Meta-analysis of the most frequent TEAEs with a frequency of≥5% in duloxetine-treated patients.

TEAEs	Included Studies(N)	OR	Heterogeneity	Effect Model	Merger value	95%CI
**Any AE**	4[12–13,15–16]	1.76	P = 0.84, I^2^ = 0%	Fixed	P<0.0001	1.36–2.28
**Nausea**	6[3,12–13,15–16]	4.72	P = 0.006,I^2^ = 70%	Random	P<0.00001	2.88–7.75
**Dry mouth**	4[12–13,15–16]	2.76	P = 0.67, I^2^ = 0%	Fixed	P = 0.0003	1.60–4.76
**Dizziness**	3[13,15–16]	2.22	P = 0.17, I^2^ = 44%	Fixed	P = 0.001	1.36–3.64
**Constipation**	4[12–13,15–16]	3.00	P = 0.96, I^2^ = 0%	Fixed	P<0.0001	1.80–4.99
**Somnolence**	4[12–13,15–16]	5.72	P = 0.23, I^2^ = 31%	Fixed	P<0.00001	2.97–11.02

TEAEs, treatment-emergent adverse events; AE, adverse events; OR, odds ratio; CI, confidence interval.

**Fig 8:**
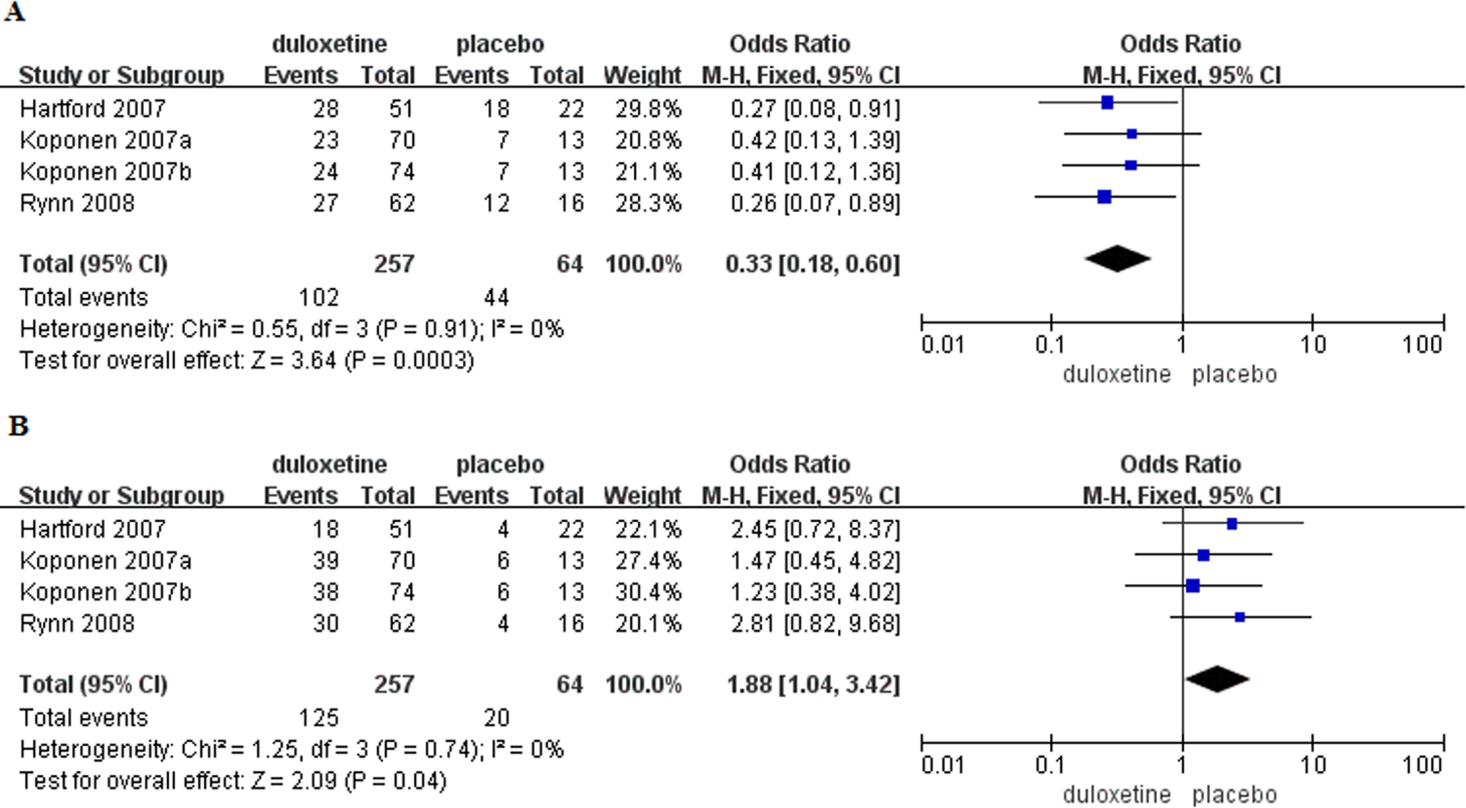
Forest plots of severity of nausea. (A) Mild nausea. (B) Moderate nausea.

Severity of nausea was reported in four trials [5,12–13]. The incidence of mild nausea was significantly higher in patients with GAD that received placebo compared to those that received duloxetine (OR = 0.33, 95%CI 0.18–0.60, P = 0.0003)(Fig 8A), while the incidence of moderate nausea was higher in patients that received duloxetine (OR = 1.88, 95%CI 1.04–3.42, P = 0.04)(Fig 8B). No patients in placebo group experienced severe nausea.

In addition to TEAEs, weight change was reported in three trials [13,15–16]. One trial reported duloxetine-treated patients had a significant weight loss compared to placebo-treated patients (MD = -1.59, 95%CI -2.24–-0.94)[13], a second trial showed mean change in weight from baseline to the end of the study was not significantly different between treatment groups (MD = -0.18, 95%CI -0.72–0.36)[15], and a third trial [16] reported no significant difference in weight loss between treatment groups in adults aged≥65 years.

## Sensitivity analysis

**Fig 9:**
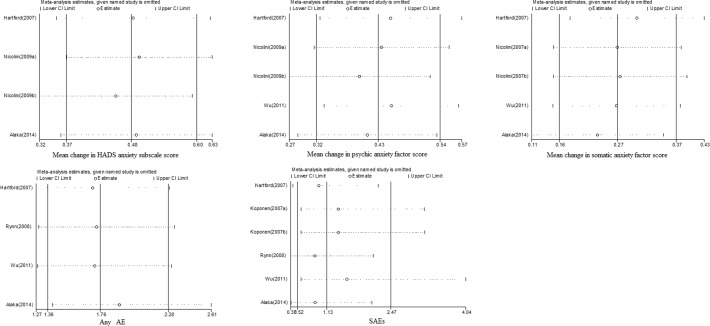
Sensitivity analyses of the main outcomes.

We conducted sensitivity analyses for the main outcomes using Stata Version 12.0 software (Fig 9) and all the results had good stability(S3 Fig).

## Discussion

This meta-analysis investigated the efficacy and tolerability of duloxetine during short-term treatment in adults with GAD. Results showed that mean changes in the HADS anxiety subscale score and HAM-A psychic anxiety factor score were significantly greater in patients with GAD that received duloxetine compared to those that received placebo, but there was no significant difference in mean change in somatic anxiety factor score. Although the discontinuation rate due to AEs, the incidence of any AE and most common TEAEs were significantly increased in patients that received duloxetine, there was no significant difference in the incidence of SAEs and therefore duloxetine was well-tolerated.

To the authors’ knowledge, this is the first meta-analysis to evaluate improvements in symptoms of psychic and somatic anxiety and the incidence of TEAEs in adults with GAD treated with duloxetine. The GAD treatment landscape is challenged by the lack of an evidence base to support clinical decision-making for treatment interventions. Selection of a pharmacologic agent is influenced by patient characteristics and the adverse events of the drug. The current meta-analysis of placebo controlled trials adds to the empirical evidence supporting a role for duloxetine in the short-term treatment of GAD and increases the quality of the database used by physicians to develop opinions about the efficacy and tolerability of duloxetine in adult patients with GAD.

Interestingly, duloxetine has been considered important in the treatment of patients with GAD that experienced concomitant chronic pain or who had significant somatic symptoms[26]. In contrast, our pooled analysis indicated duloxetine was particularly effective for symptoms of psychic anxiety. Additional high-quality trials with larger sample sizes are warranted to clearly define the efficacy and tolerability of duloxetine in GAD.

Previously, mixed treatment meta-analyses were used to explore the comparative efficacy and tolerability of treatments for GAD, whereby duloxetine was superior to venlafaxine (SNRI) and pregabalin (anticonvulsant) in terms of clinical response during the initial 8-week treatment phase. However, duloxetine was ranked last in terms of withdrawals because of AEs [27–28]. With regard to cost-effectiveness, duloxetine was more cost-effective than pregabalin but less cost-effective than sertraline (SSRI) and venlafaxine [28].

The current analysis had several strengths. First, the included studies were randomized, double-blind, placebo-controlled trials. Second, we performed manual searches of the reference lists for all relevant articles and contacted the corresponding authors of some RCTs for missing information. Third, we set strict inclusion criteria and GAD severity at entry was assessed using the HADS anxiety subscale rather than the HAM-A, avoiding potential for inflation of the HAM-A total scores at baseline. However, the analysis was associated with some limitations. First, imposing no restriction on fixed or flexible dose of duloxetine increased heterogeneity between the trials included in the analysis of tolerability. We attempted to overcome this limitation using sensitivity analyses that showed the results were robust. Second, some trials were excluded as appropriate data could not be extracted and missing information could not be obtained from study authors. Third, we utilized a funnel plot to assess potential publication bias; generally, funnel plots should only be used to assess publication bias in reviews that include≥10 studies, and even then researchers may be misled by their shape[29–30].

In conclusion, the findings of our meta-analysis suggested that duloxetine resulted in a greater improvement in symptoms of psychic anxiety and similar changes in symptoms of somatic anxiety compared to placebo, with acceptable tolerability, during short-term treatment of adults with GAD.

## Supporting information

S1 File Study protocol. (DOC)

S2 File PRISMA 2009 checklist. (DOC)

S3 File Detailed search strategy. (DOC)

S1 Table Publication bias assessment of all the outcomes. (DOC)

S1 Fig Forest plots of response and remission rates. (TIF)

S2 Fig Forest plot of discontinuation rate due to adverse events. (TIF)

S3 Fig Sensitivity analyses of the remaining outcomes. (TIF)
