# Supplementary material for: Efficacy and tolerability of short-term duloxetine treatment in adults with generalized anxiety disorder: A meta-analysis
Source: PLoS One. 2018 Mar 20;13(3):e0194501. doi: 10.1371/journal.pone.0194501 (PMC5860757; doi:10.1371/journal.pone.0194501)
Supplement: S1 File — (DOC) [file pone.0194501.s001.doc]

**Efficacy and tolerability of short-term duloxetine treatment in adults with generalized anxiety disorder: A meta-analysis protocol**

Xinyuan Li^1¶^, Lijun Zhu^2¶^，Chunkui Zhou^1¶^, Jing Liu^1^，Heqian Du^1^, Chenglin Wang^1^, Shaokuan Fang^1^*

**Review question(s)**

Although efficacy of duloxetine for generalized anxiety disorder (GAD) has been reported in previous analysis in 2016, the issue that improvement in psychic and somatic symptoms and treatment-emergent adverse effects in adults during short-term treatment has not been evaluated.

**Searches**

Literature databases include Pubmed, Embase, Cochrane Central Register of Controlled Trials, Web of science and clinical trials. We conduct manual searches of the reference lists of relevant articles.

Contact of experts: we attempt to contact the authors of relevant articles when needed. There were no restrictions on the date of publication. Language was limited to English.

**Type of study to be included:**

Study designs: experimental (randomized controlled trials).

**Participants/ population**

Patients aged or older than 18 years meeting the DSM-IV criteria for GAD

**Exposure(s)**

Duloxetine or duloxetine plus antipsychotics

**Comparator(s)/ control**

Placebo-controlled

**Outcome(s)**

Efficacy outcome: assessed by the Hospital Anxiety and Depression Scale(HADS) anxiety subscale score and the Hamilton Rating Scale for Anxiety(HAM-A) psychic and somatic anxiety factor scores;

Tolerability outcome: assessed by the incidence of treatment emergent adverse effects(TEAEs).

**Data extraction, (selection and coding)**

Selection process:

Title and abstract screening: Teams of two reviewers will use the above eligibility criteria to screen titles and abstracts of identified citations in duplicate and independently for potential eligibility. We will get the full text for citations judged as potentially eligible by at least one of the two reviewers.

Full-text screening: Teams of two reviewers will use the above eligibility criteria to screen the full texts in duplicate and independently for eligibility. The teams of two reviewers will resolve disagreement by discussion or with the help of a third reviewer.

We will use standardized and pilot tested screening forms. We will conduct calibration exercises to ensure the validity of the selection process.

Data abstraction process:

Teams of two reviewers will abstract data from eligible studies in duplicate and independently. They will resolve disagreements by discussion or with the help of a third reviewer.

We will collect the following data: the first author’s name, year of publication, age, sex distribution, sample, study design, patient population, treatment duration, intervention, outcomes.

We will use standardized and pilot tested data abstraction forms.

We will conduct calibration exercises to ensure the validity of the data abstraction process.

**Risk of bias (quality) assessment:**

Teams of two reviewers will assess the risk of bias in each study in duplicate and independently. They will resolve disagreements by discussion or with the help of a third reviewer.

We will use the Cochrane Risk of Bias tool to assess the risk of bias in randomized trials.

We will calculate the risk of bias using the following criteria:

The likelihood of risk of bias included random sequence generation, allocation concealment, blinding of outcome assessment, blinding of participants and personnel, incomplete outcome data, selective reporting and other bias.

We will grade each potential source of bias as high, low or unclear risk of bias. We will use unclear when the authors did not report enough information for us to make the judgment.

We will not exclude any study based on quality.

**Strategy for data synthesis**

We will conduct a meta-analysis to pool the results across studies for duloxetine as the exposure of interest, and ‘efficacy and tolerability’ as the outcome of interest.

We will carry out statistical analysis using RevMan(version 5.3). For Dichotomous data, we will calculate the ORs for each study. For continuous data, we will calculate the mean difference for each study.

We will test the results for homogeneity using the I^2^ test and considered heterogeneity present if I^2^≥50% and P<0.05. We will conduct the sensitivity analysis.

We will assess publication bias using the funnel plot and the Begg’s/Egger’s test via Stata Version 12.0 software.

**Dissemination plans**

We will publish results in international, peer-reviewed journals.
