# Supplementary material for: Efficacy and tolerability of short-term duloxetine treatment in adults with generalized anxiety disorder: A meta-analysis
Source: PLoS One. 2018 Mar 20;13(3):e0194501. doi: 10.1371/journal.pone.0194501 (PMC5860757; doi:10.1371/journal.pone.0194501)
Supplement: S3 File — (DOC) [file pone.0194501.s003.doc]

Search strategy for PubMed

#1 ((cymbalta) OR LY248686) OR duloxetine—2260

#2 (GAD) OR generalized anxiety disorder—13934

#3 randomized controlled trials—590760

#1 AND #2 AND #3 ((((GAD) OR generalized anxiety disorder)) AND randomized controlled trials) AND (((cymbalta) OR LY248686) OR duloxetine)—32

Search strategy for Web of Science

(((generalized anxiety disorder OR GAD) AND ((duloxetine OR LY248686) OR cymbalta)) AND (randomized controlled trials))—70

Search strategy for Embase

#1 'duloxetine'/exp—9131

#2 'generalized anxiety disorder'/exp—8758

#3 #1 AND #2—352

#3 AND ('controlled clinical trial'/de OR 'controlled study'/de OR 'randomized controlled trial'/de OR 'randomized controlled trial (topic)'/de)—110

Search strategy for Cochrane Center Register of Controlled Trials(CENTRAL)

(duloxetine OR LY248686 OR cymbalta) AND (generalized anxiety disorder OR GAD) AND (randomized controlled trials)—38

Search strategy for ClinicalTrials database

(duloxetine OR LY248686 OR cymbalta) AND (generalized anxiety disorder OR GAD)—13
