# Supplementary material for: Efficacy and tolerability of short-term duloxetine treatment in adults with generalized anxiety disorder: A meta-analysis
Source: PLoS One. 2018 Mar 20;13(3):e0194501. doi: 10.1371/journal.pone.0194501 (PMC5860757; doi:10.1371/journal.pone.0194501)
Supplement: S1 Table — (DOC) [file pone.0194501.s004.doc]

**Table S1. Publication bias assessments of all the outcomes.**

| **Outcomes** | **Egger’s test**  **(t-value)** | **P-value** |
| --- | --- | --- |
| **Baseline HADS anxiety subscale score** | 1.01 | 0.370 |
| **Mean change in HADS anxiety subscale score** | 2.40 | 0.138 |
| **Baseline psychic anxiety factor score** | -0.43 | 0.685 |
| **Mean change in psychic anxiety factor score** | 1.81 | 0.167 |
| **Baseline somatic anxiety factor score** | -0.18 | 0.865 |
| **Mean change in somatic anxiety factor score** | 0.04 | 0.974 |
| **Any AE** | -7.71 | 0.016 |
| **Nausea** | 1.39 | 0.236 |
| **Mild nausea** | 2.93 | 0.100 |
| **Moderate nausea** | -14.97 | 0.004 |
| **Dry mouth** | -0.91 | 0.459 |
| **Dizziness** | 0.04 | 0.973 |
| **Constipation** | 11.39 | 0.008 |
| **Somnolence** | -0.33 | 0.773 |
