# Supplementary figures and images for: Efficacy and tolerability of short-term duloxetine treatment in adults with generalized anxiety disorder: A meta-analysis
Source: PLoS One. 2018 Mar 20;13(3):e0194501. doi: 10.1371/journal.pone.0194501 (PMC5860757; doi:10.1371/journal.pone.0194501)

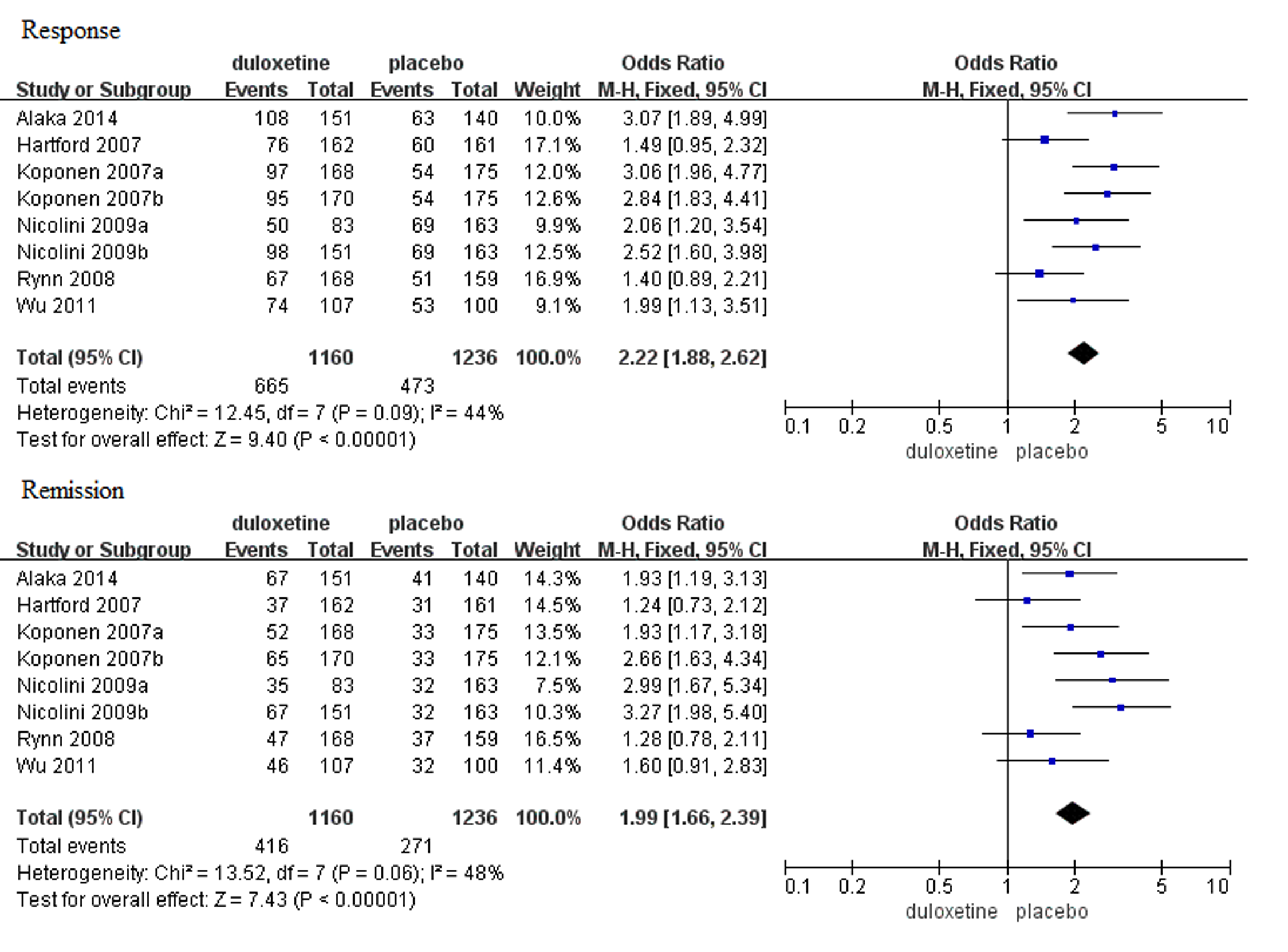

Supplement: S1 Fig — (TIF) [file pone.0194501.s005.tif]

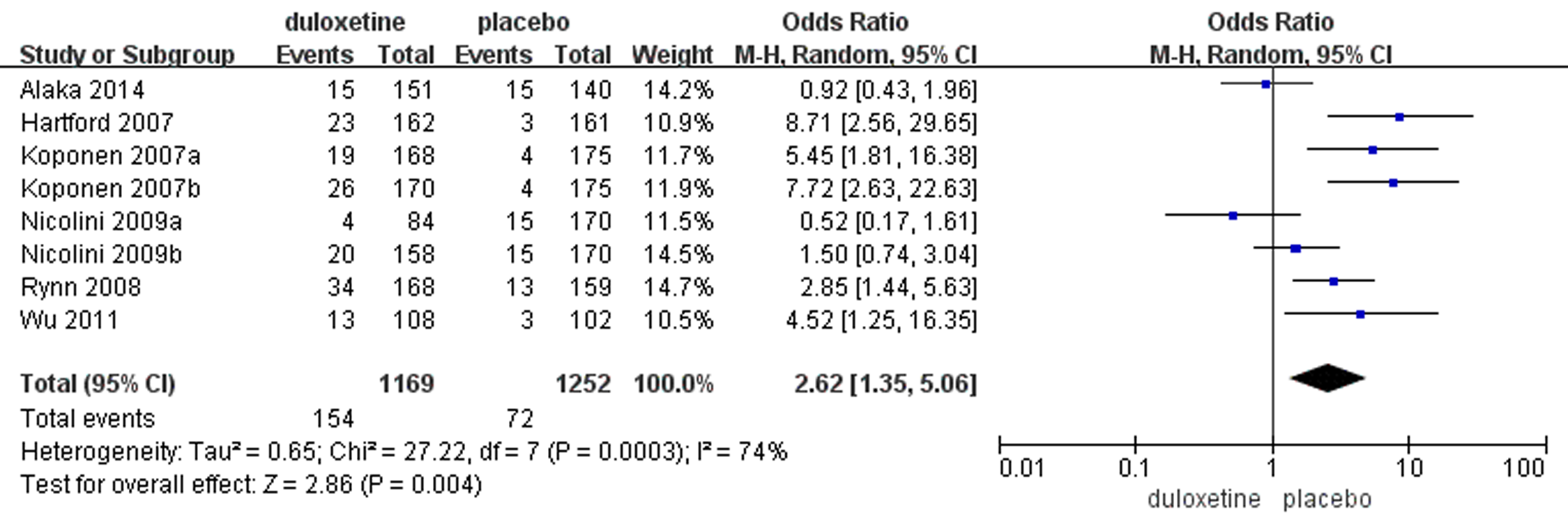

Supplement: S2 Fig — (TIF) [file pone.0194501.s006.tif]

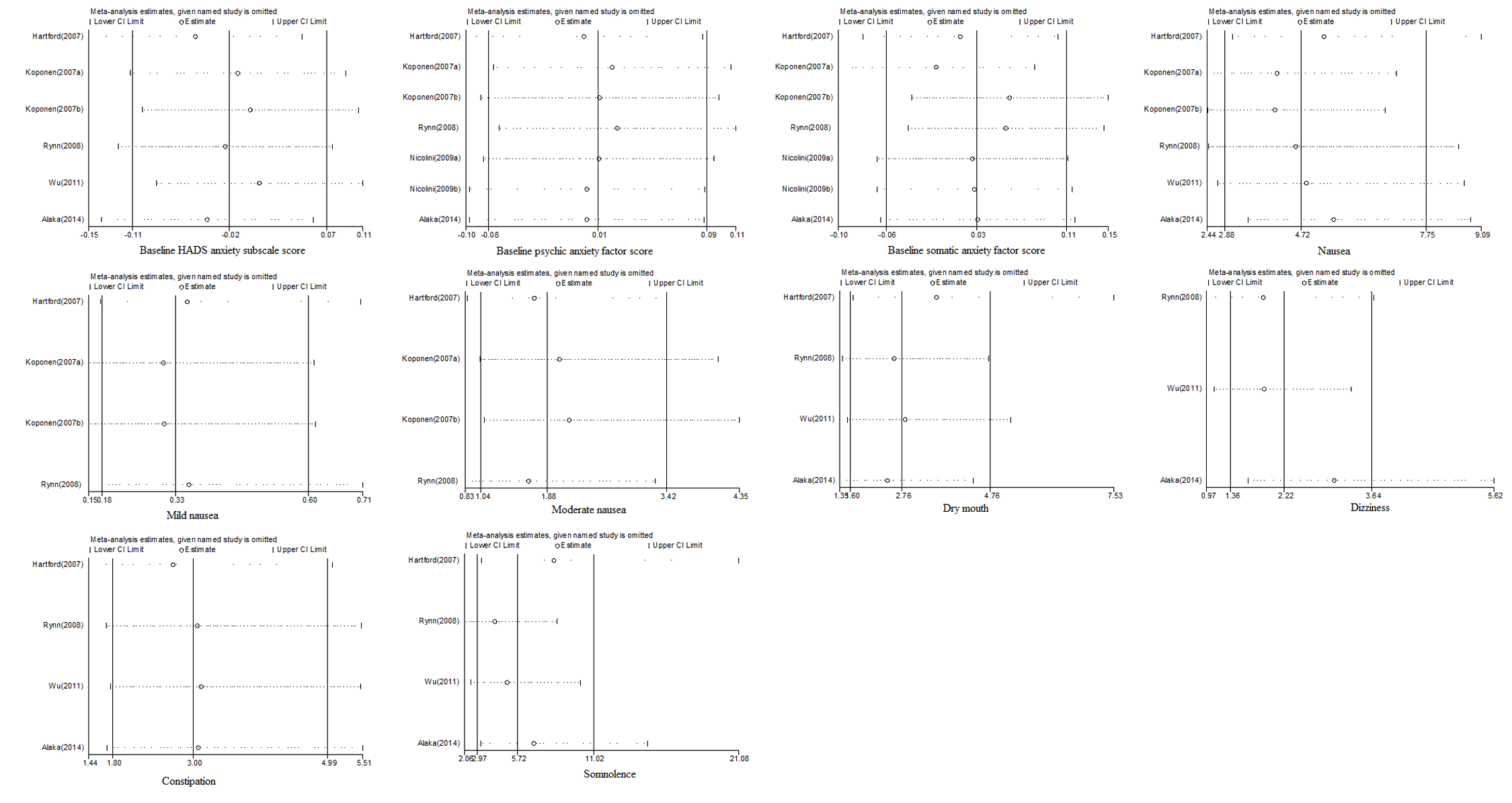

Supplement: S3 Fig — (TIF) [file pone.0194501.s007.tif]
